# Effect of short inter-pregnancy interval on perinatal outcomes among pregnant women in North-west Ethiopia: A prospective cohort study

**DOI:** 10.3389/fpubh.2022.953481

**Published:** 2022-08-08

**Authors:** Leta Gurmu, Negash Wakgari, Tufa Kolola, Kababa Temesgen Danusa

**Affiliations:** ^1^Department of Midwifery, College of Health Science, Assosa University, Assosa, Ethiopia; ^2^Department of Midwifery, College of Medicine and Health Sciences, Ambo University, Ambo, Ethiopia; ^3^Department of Public Health, College of Medicine and Health Sciences, Ambo University, Ambo, Ethiopia

**Keywords:** effect, optimal birth interval, perinatal outcomes, short inter-pregnancy interval, Ethiopia

## Abstract

**Background:**

Inter-pregnancy interval (IPI) is the elapse of time between the end of one pregnancy and the conception of another pregnancy, while birth to pregnancy interval, is the time gap between live birth and the conception of the next pregnancy. Hence, this study assessed the effects of short inter-pregnancy intervals on perinatal outcomes among women who gave birth in public health institutions of Assosa zone, North-west Ethiopia.

**Methods:**

An institution-based prospective cohort study was conducted among 456 mothers who visited health facilities for the fourth antenatal care appointment (152 exposed and 304 non-exposed). Women who gave their recent birth with the pregnancy interval of <24 months or/and had an abortion history of <6 months were considered as exposed otherwise non-exposed. Data was collected through face-to-face interviews by using questionnaires and checklists. The collected data was entered using Epi-data and exported to STATA for analysis. A log-binomial regression model was used to identify the effect of short inter-pregnancy intervals on the perinatal outcomes.

**Results:**

The overall incidence of adverse perinatal outcomes is 24%. Mothers who had short inter-pregnancy intervals have two times the risk to develop low birth weight (RR: 2.1, 95%CI: 1.16–3.82), and low Apgar score (RR: 2.1, 95%CI: 1.06–2.69). Similarly, the risk to develop small for gestational age (RR: 2.6, 95% CI: 1.19–7.54), and preterm birth (RR: 3.14, 95%CI: 1.05–4.66) was about 3 times among mothers who had short inter-pregnancy interval compared to mothers who had an optimal inter-pregnancy interval.

**Conclusion:**

Short inter-pregnancy interval increases the risk of low birth weight, preterm birth, small for gestational age, and low Apgar score. Health Policy makers, National health managers and health care providers should work on increasing the awareness of optimal inter-pregnancy intervals and postpartum family planning utilization to reduce the effect of short inter-pregnancy intervals on adverse perinatal outcomes.

## Introduction

The World Health Organization (WHO) technical consultation group recommended an optimal interval of birth to the next pregnancy a minimum of 24 months interval or birth to a birth interval of 33 months or more months in two consecutive births (1). Inter-pregnancy interval (IPI) is the elapse of time between the end of one pregnancy and the conception of another pregnancy, while birth to pregnancy interval, is the time gap between live birth and the conception of the next pregnancy (2).

The need to pay attention to birth interval is evidence-based and the adverse outcomes of closely spaced births are enormous (3, 4). Inter-pregnancy intervals of <18 months have significantly increased the risk of adverse perinatal outcomes (5). Optimal birth intervals have a reduced risk of various adverse perinatal and maternal outcomes, such as low birth weight, preterm birth, small for gestational age babies, complications of pregnancy, and maternal mortality (4, 5). Extremes of the interpregnancy interval have been identified as a risk factor for different adverse perinatal outcomes, such as preterm birth, low birth weight, and perinatal death (4–6).

Globally, more than 2.5 million perinatal deaths occur annually, of which 95% take place in developing countries in which short inter-pregnancy interval is independently associated with a high risk of adverse perinatal outcomes (6). Similarly, in sub-Saharan African countries numerous adverse perinatal outcomes were reported due to short interpregnancy intervals (7, 8). Adverse perinatal outcomes such as stillbirth, low birth weight, preterm birth, and small for gestational age constituted the highest rates of adverse pregnancy outcomes and are common in developing countries (9).

The study in Tanzania showed that the incidence of preterm birth, low birth weight, and perinatal death were 12.57, 11.97, and 4.14%, respectively, among mothers who had a short inter-pregnancy interval (10). Additionally, another study was done in central Tanzania reported that mothers having short inter-pregnancy intervals had more risk to develop adverse perinatal outcomes which imply, the incidence of low birth weight 26.6%, small for gestational age 23.3%, and preterm birth of 29.3% (11). Ethiopian demographic and health surveys report that one in 35 children die within the first month and early neonatal death is 72 per thousand deliveries among mothers who had a short inter-pregnancy interval (12). Similarly, the previous studies conducted in Ethiopia imply that there is an increased risk of adverse pregnancy outcomes among mothers with short inter-pregnancy intervals (13–15). Though many studies were conducted on short inter-pregnancy intervals focusing on the adverse maternal outcome, there is a lack of studies focusing on the effects of short inter-pregnancy intervals on perinatal outcomes to inform policymakers. Therefore, this study aimed to assess the effects of short inter-pregnancy intervals on perinatal outcomes among women who gave birth in public health institutions of Assosa zone, Benishangul Gumuz, North Western, Ethiopia.

## Methods

### Study setting, design, and population

Facility-based a prospective cohort study was conducted in Assosa Zone from March to July 2020. Assosa zone is one of the three zones found in Benishangul Gumuz regional state, North-west Ethiopia located 676 Km to the West of Addis Ababa, the capital city of Ethiopia. Based on the 2007 census, this zone has an estimated of 450,000 total population. Assosa zone consists of 20 government health institutions: one general hospital, one district hospital, and 18 public health centers providing delivery services during the study period (12).

Selected mothers who come for ANC visit in the last trimester of pregnancy or after 28 weeks of gestational age up to 1 week after delivery, in the selected health institutions were the study population. The classification of exposed and non-exposed was determined based on inter-pregnancy interval. Accordingly, women with an inter-pregnancy interval of <24 months, or/and who had an abortion history of <6 months were exposed, while those women with an inter-pregnancy interval of ≥24 months or/ and had an abortion history of ≥6 months were categorized as non-exposed. Mothers with a singleton pregnancy and conceived at least for the second time were included in the study. Those who are unable to respond and had medical illnesses such as hypertensive disorders, antepartum hemorrhage, and diabetes mellitus, and had unknown last normal menstrual period and missed early ultrasound reports from the chart are excluded from the study during selection process.

### Sample size determination and sampling procedure

A double population proportion formula was used to determine sample size using open EPI info version 7.2 with the following assumptions: power 80%, 95% confidence level, a ratio of non-exposed to exposed 2:1 and the proportion of preterm birth among exposed 10.4%, and percentage of outcome among non-exposed 2.9% taken from a previous study conducted in Northern Ethiopia (13). After adding 10% of the loss to follow-up the final sample size became 456 with 152 exposures and 304 non-exposures.

A simple random sampling technique was used to select five public Health institutions in the Assosa zone: one general hospital, one dsistricthospital, and three health centers. The sample size was allocated to each hospital and health center based on the previous year's (2019) antenatal care performance reports (mothers come for ANC visit in the last trimester pregnancy or after 28 weeks of gestational age up to 1 week after delivery). As a result, a total of 1,284 pregnant mothers have visited five health institutions for ANC in the same month of the previous year of the study. The k^th^-value is calculated by dividing 1,284/456 = 3. A systematic random sampling method was used to select both exposed and non-exposed pregnant mothers from each health institution using every 3rd interval until the expected sample was obtained. Eventually, a total of 456 (152 exposed and 304 non-exposed) were selected and enrolled in the study depending on proposition population size (PPS) according to case flow of the selected health facilities.

### Data collection tool and techniques

The data was collected through a pre-tested and structured interviewer-administered questionnaire and checklist. The data collection tool was adapted and modified from previous similar studies (10, 13, 16). The tool has three sections: socio-demographic, obstetric, and reproductive health history. Gestational age was estimated as the interval of complete weeks from the last normal menstrual period to the child's date of birth. When there are extra days it is counted to the near lowest gestational age but if the mother did not remember or recall her last normal menstrual period; gestational age was estimated from the ultrasound result through reviewing the mother's chart. Then the mothers, who were eligible for the study were categorized into exposed and non-exposed based on pregnancy intervals. The study was followed Starting from 28 GA weeks up to 1 week after delivery to seek the effect of inter-pregnancy interval on perinatal outcomes. Five BSc midwives were assigned to each health institution to gather data in two phases. Two MSc holders were recruited to supervise the overall data collection technique. During the first phase of data collection; socio-demographic and obstetric characteristics were collected and follow-up. The second phase of data collection was held at the labor and delivery ward to assess perinatal outcomes.

### Data quality control

Data quality was ensured during data collection, coding, entry, and analysis. The training was given to data collectors and supervisors before data collection to prevent any confusion and have a common understanding of the study. Before actual data collection, a pre-test was done by using 5% of the sample size in non-selected health institutions to check the validity and reliability of the questionnaire and evaluate the outcome of the tool. Completeness of data was checked daily and coded before data entry.

### Data processing and analysis

The collected data were entered and cleaned using Epi-data version 3.1 and exported to STATA version 14 softwarefor data analysis. Basic descriptive analyses were done and presented as frequency and percent for categorical variables. Continuous variables were reported using mean with standard deviation. To estimate relative risk, a log-binomial regressions model by adding “form on the command” was used to identify the effect of short inter-pregnancy intervals on perinatal outcomes. A *p*-value <0.05 with 95% CI was considered to declare statistical significance. Maternal age, educational status, residence, gravidity, parity, maternal hemoglobin status, and maternal Rh-factor were controlled in the statistical models.

### Operational and term definitions

Perinatal period: the period starting from 28 weeks gestational age and ends 1 week after delivery. Short inter-pregnancy interval: Inter-pregnancy interval of <24 months from the date of live birth to the conception of the subsequent pregnancy, while an optimal inter-pregnancy interval is a pregnancy interval of 24 months and above from the date of live birth to the conception of the subsequent pregnancy. Adverse perinatal outcome: the adverse perinatal outcome was measured if the following conditions in current pregnancies occurred. These include women who gave birth to low birth weight, low Apgar score, preterm birth, perinatal mortality, and small for gestational age. Small size for gestational age: Birth weight below the 10th percentile for the infant's gestational age. Preterm birth: Neonate born before 37 completed weeks of gestation. It was measured by using the last menstrual period, early ultrasound result, or Ballard maturity examination. Perinatal mortality: Loss of a baby before or during delivery after 28 weeks gestational age within 7 days of life. Low Apgar score: APGAR score < 7 at first, and fifth minutes of life. Low birth weight: Birth weight below 2,500 grams (17, 18).

### Ethics statement

Ethical clearance was obtained from the Ambo University, College of Medicine and Health Science ethical review committee with the reference number PGC/83/2020. Then the official letter was submitted to selected health institutions. The permission and agreement consent was obtained from the selected health institutions. Informed written consent was obtained from the study participants and guardians for those who were under 18 years old. Throughout the study, participants were informed that data was kept private and confidential and used only for research purposes. The participants were also assured that they have the right to refuse or withdraw if they are not comfortable at any time.

## Results

### Socio-demographic characteristics

Four hundred thirty-eight (438) courts of pregnant mothers participated from 456 pregnant mothers, who were followed with 18(3.9%) lost to follow-up. The mean age of the mothers was 28 years (SD ± 6.28) with the age range of 15–48 years. The majority 409(93.4%) of mothers were married (Table 1).

**Table 1:** Socio-demographic characteristics of mothers in Assosa zone public health facility, Northern West, Ethiopia, 2020 (*N* = 438).

**Variables**	**Short IPI,** ***N* (%)**	**Optimal IPI,** ***N* (%)**
**Age in complete years (438)**		
15–24	16 (29.1)	39 (71.9)
25–34	94 (39)	147 (61)
35–48	36 (25.4)	106 (74.6)
Total	146 (33.3)	292 (66.7)
**Marital status**		
Married	131 (32)	278 (68)
Divorced	0 (0.0)	5 (100)
Widowed	15 (62.5)	9 (37.5)
Total	146 (33.3)	292 (66.7)
**Maternal education**		
Unable to read and write	26 (22.8)	88 (87.2)
Able to read and write	0 (0)	61 (100)
Elementary school (1–8)	71 (47)	80 (53)
High school (9–10)	31 (38.8)	49 (61.3)
Diploma and above	18 (56.3)	14 (43.7)
Total	146 (33.3)	292 (66.7)
**Maternal occupation**		
Housewife	88 (35.6)	159 (64.4)
Farmer	22 (33.3)	44 (66.7)
Civil servant	17 (23.9)	54 (76.1)
Businesswoman	19 (35.2)	35 (64.8)
Total	146 (33.3)	292 (66.7)
**Residences**		
Urban	102 (32.8)	209 (67.2)
Rural	44 (34.6)	83 (65.4)
Total	146 (33.3)	292 (66.7)

### Obstetrics characteristics of mothers

Among pregnant mothers who were attending the public facility of Assosa zone majority of the 89% initiated ANC follow-up after 24–32 weeks and followed by 16 weeks. With concern to parity, 81.3% of the mothers were multipara and 33 of them were experienced short inter-pregnancy intervals. Thirteen percent of mothers have <11 g/dl of hemoglobin concentration (Table 2).

**Table 2:** Obstetrics characteristics of mothers in Assosa zone public health facility, North West Ethiopia, 2020 (*N* = 438).

**Variables**	**Short IPI (*n* = 146)**	**Optimal IPI (*n* = 292)**
	**Frequency (%)**	**Frequency (%)**
**Gravidity**		
≤4 pregnancy	78 (30.5)	178 (69.5)
>4 pregnancy	68 (37.4)	114 (63.6)
Total	146 (33.3)	292 (66.7)
**Parity**		
Grand multipara	22 (26.8)	60 (73.2)
Multipara	124 (38.8)	232 (65.2.)
Total	146 (33.3)	292 (66.7)
**Initiation of ANC**		
Within 16 weeks	4 (15.4)	22 (94.6)
24-28 weeks	93 (34.6)	176 (65.4)
28–32 weeks	27 (22.3)	94 (77.7)
32–36 weeks	22 (100)	0 (0.0)
Total	146 (33.3)	292 (66.7)
**Iron supplementation**		
No	14 (26.9)	256 (73.1)
Yes	132 (34.2)	36 (65.8)
Total	146 (33.3)	292 (66.7)
**Rh factors**		
Negative	18 (40)	27 (60)
Positive	128 (32.6)	265 (67.4)
**Maternal hemoglobin**		
<11 mg/Dl	19 (33.3)	38 (66.7)
≥11 mg/Dl	127 (33.3)	254 (66.7)

### Incidence of adverse perinatal outcome

The overall incidence among cases (short IPI) was 333 per 1,000 live birth. The incidence of low birth weight was higher among newborn babies delivered from women with short IPI than mothers with optimal IPI 136.9/1,000 vs. 65/1,000 live birth. Similarly, the incidence of low Apgar score was higher among newborn babies delivered from women with short IPI than women with optimal IPI 116/1,000 vs. 106/1,000 live birth.

The mean birth weight of babies born from women with short IPI was 2,347 and 3,275 grams among mothers with optimal IPI. In addition, 62/1,000 live birth from women with short IPI and 21/1,000 from women with optimal IPI have had small gestational age. Additionally, higher preterm births occurred among pregnant women with short IPI than optimal IPI with 55 per 1,000 vs. 48 per 1,000 live births. that mothers with a Short IPI had higher risk for lower gestational age at delivery (62 per 1,000 live birth), compared to those with Optimal IPI920.5 per 1,000 live birth).

Perinatal mortality occurred in 14 per 1,000 live birth of women with short IPI and 7 per 1,000 live births among mothers with optimal IPI, respectively. Moreover, the incidence of preterm birth is higher among women with short IPI compared to those who had optimal IPI with 75 and 45 per 1,000 live births, respectively.

### Risk of adverse perinatal outcome associated with inter-pregnancy interval

The risk of having a low birth weight baby (RR: 2.1, 95%CI: 1.16–3.82) and low Apgar score (RR: 2.1, 95%CI: 1.06–2.69) was two times more likely among mothers with short IPI than those mothers with optimal IPI. Similarly, the risk of small for gestational age was about three times among mothers with short IPI (RR: 2.6, 95%CI: 1.19–7.54) compared to mothers with optimal inter-pregnancy intervals. The risk to develop preterm babies was 3 times among mothers with short IPI (RR: 3.14, 95% CI: 1.05–4.66) than mothers with optimal IP interval (Table 3).

**Table 3:** Association between IPI and adverse perinatal outcome among pregnant mothers 2020 (*N* = 438).

**Variables**	**Short IPI,** ***n* = 146**	**Optimal IPI,** ***n* = 292**	**RR**	**95% CI for RR**	***P*-value**
**Low birth weight**					
Yes	20 (13.69%)	19 (6.5%)	2.1054	**1.16–3.82**	**0.014**
No	126 (86.31%)	273 (93.5%)			
**Low APGAR score**					
Yes	17 (11.6%)	31 (10.6%)	1.0967	**1.06**–**2.69**	**0.034**
No	129 (88.4%)	261 (89.4%)			
**Perinatal mortality**					
Yes	2 (1.4%)	2 (0.7%)	2	0.28–14.05	0.486
No	144 (98.6%)	290 (99.3%)			
**Small for gestational age**					
Yes	9 (6.2%)	6 (2.05%)	2.6667	**1.19**–**7.54**	**0.006**
No	137 (93.8%)	286 (97.95%)			
**Preterm birth**					
Yes	11 (7.5%)	13 (4.5%)	3.1428	**1.04**–**4.66**	**0.017**
No	138 (92.5%)	278 (95.5%)			

## Discussion

In this study, the overall incidence of adverse perinatal outcomes was 24% [95% CI: 20.1–28.1]. This finding is in line with the studies done in Denmark 22.4%, Tanzania 21%, and Ethiopia (19–22). The rationale of this could be mothers with short inter-pregnancy intervals might not recover from physiological change happen during pregnancy and after delivery which imposes in diminution of macro and micro-nutrients, abnormal remodeling of endometrial blood vessels, anemia and increasing the risks of certain other factors induces adverse perinatal outcomes (23, 24).

The incidence of low birth weight was 13.96 and 6.5% among short and optimal inter-pregnancy intervals, respectively. This finding is lower than the study conducted in Egypt (25). This might be due to a reduction in placental blood flow which would affect the exchange of nutrients and oxygen between the mother and the fetus. The woman who had short IPI had 2 times the risk to develop low birth weight than their counterpart. This is in line with the study conducted in Tanzania (10).

The incidence of small for gestational age was 6.2 and 2.05% or (62 and 20.5) per 1,000 live births among short and optimal inter-pregnancy intervals, respectively. This finding is higher than the study conducted in Bangladesh (26). This discrepancy is might be due to the socio-demographic and cultural diversity of the mothers. Short IPI is associated with low hemoglobin levels which might have an effect on the oxygen-bearing capacity for the placenta and consequently decrease oxygen supply for the fetus in the womb (10, 26). Women who had short IPI were 3 times riskier to have a small gestational age baby than optimal interpregnancy interval. This finding is higher than the study conducted in Canada (27). The difference might be due to variation in socio-economic status and low access to quality health service developing world.

In this study, the risk of developing preterm birth is 3 times more likely among short IPI than mothers who had an optimal inter-pregnancy interval. This finding is consistent with the study done in Australia (28). This may be because the woman who had short IPI is prone to develop APH and cervical incompetency which results in preterm birth than the woman with optimal inter-pregnancy interval (29).

The incidence of the low Apgar score is 11.6 and 10.6% among short and optimal inter-pregnancy intervals, respectively. The finding of this study is higher than the study done in Nigeria (30). A possible explanation might be mothers who experience short IPI may be physiologically not well-recovered and fetoplacental blood follow is compromised consequently the fetus may not tolerate labor and develop intrapartum asphyxia (31). Women who had short inter-pregnancy intervals were more at risk to develop a low Apgar score than the optimal inter-pregnancy interval. This finding is in line with the study conducted in Kenya (32, 33).

## Conclusions

This study indicates that a short inter-pregnancy interval increases the risk of low birth weight, preterm birth, small for gestational age, and low Apgar score. However, short inter-pregnancy does not affect perinatal mortality. Hence, Health Policy makers, Researchers, National health managers and health care providers should work on increasing the awareness of optimal inter-pregnancy intervals and postpartum family planning utilization to reduce the effect of short inter-pregnancy intervals on adverse perinatal outcomes. Expectant women should be fully informed about the risk of short inter-pregnancy intervals on perinatal outcomes.

### Limitations of the study

First, there was a loss of follow-up of participants. Secondly, since this study was conducted in one zone, the finding might not be generalized to the entire Region. Lastly, for those mothers whose last normal menstrual period is unknown and early ultrasound report is missed in her chart, was not possible to enroll and difficult to estimate their gestational age.

## Data availability statement

The raw data supporting the conclusions of this article will be made available by the authors, without undue reservation.

## Ethics statement

The studies involving human participants were reviewed and approved by Ambo University, College of Medicine and Health Science Ethical Review Committee with the reference number PGC/83/2020. Written informed consent to participate in this study was provided by the participants' legal guardian/next of kin.

## Author contributions

LG, NW, TK, and KD conceived the study and were involved in the study design, reviewed the article, analysis, report writing, and drafted the manuscript. All authors have read and approved the final manuscript.

## Conflict of interest

The authors declare that the research was conducted in the absence of any commercial or financial relationships that could be construed as a potential conflict of interest.

## Publisher's note

All claims expressed in this article are solely those of the authors and do not necessarily represent those of their affiliated organizations, or those of the publisher, the editors and the reviewers. Any product that may be evaluated in this article, or claim that may be made by its manufacturer, is not guaranteed or endorsed by the publisher.

## Abbreviations

ANC: Antenatal care
BTP: Birth to Pregnancy
CSA: Central Statistics Agency
FMOH: Federal Ministry of Health
IPI: Inter-Pregnancy Interval
SIPI: Short Inter-Pregnancy Interval
OIPI: Optimal Inter-Pregnancy Interval
LBW: Low Birth Wight
OR: Odds Ratio.
